# Supplementary material for: Global environmental implications of atmospheric methane removal through chlorine-mediated chemistry-climate interactions
Source: Nat Commun. 2023 Jul 8;14:4045. doi: 10.1038/s41467-023-39794-7 (PMC10329632; doi:10.1038/s41467-023-39794-7)
Supplement: Supplementary file 1 — Supplementary Information [file 41467_2023_39794_MOESM1_ESM.pdf]

Supplementary information for

Global environment implications of atmospheric methane removal through chlorine-mediated chemistry-climate interactions

Qinyi Li[1,2,#], Daphne Meidan[3,#], Peter Hess[4], Juan A. Añel[5,1], Carlos A. Cuevas[1], Scott Doney[6], Rafael P. Fernandez[7], Maarten van Herpen[8], Lena Höglund-Isaksson[9], Matthew S. Johnson[10], Douglas E. Kinnison[11], Jean-François Lamarque[12], Thomas Röckmann[13], Natalie M. Mahowald[3,*], and Alfonso Saiz-Lopez[1,*]

[1] Department of Atmospheric Chemistry and Climate, Institute of Physical Chemistry Blas Cabrera, CSIC, Madrid 28006, Spain;

[2] Department of Civil and Environmental Engineering, The Hong Kong Polytechnic University, Hong Kong 999077, China;

[3] Department of Earth and Atmospheric Sciences, Atkinson Center for a Sustainable Future, Cornell University, Ithaca, NY, USA;

[4] Department of Biological and Environmental Engineering, Cornell University, Ithaca, NY, USA;

[5] EPhysLab, CIM-Uvigo, Universidade de Vigo, Ourense, Spain;

[6] Department of Environmental Sciences, University of Virginia, Charlottesville, VA, USA;

[7] Institute for Interdisciplinary Science (ICB), National Research Council (CONICET), FCEN-UNCuyo, Mendoza, Argentina;

[8] Acacia Impact Innovation BV, Acacialaan 9, 5384 BB, Heesch, The Netherlands;

[9] Air Quality and Greenhouse Gases Program (AIR), International Institute for Applied Systems Analysis (IIASA), 2361 Laxenburg, Austria;

[10] Department of Chemistry, University of Copenhagen, Universitetsparken 5, DK-2100 Copenhagen Ø, Denmark

[11] Atmospheric Chemistry Observations & Modeling Laboratory, National Center for Atmospheric Research, Boulder, CO, USA;

[12] Climate and Global Dynamics Laboratory, National Center for Atmospheric Research, Boulder, CO, USA.

[13] Institute for Marine and Atmospheric Research Utrecht, Utrecht University, Princetonplein 5, 3584CC, Utrecht, the Netherlands

[#] These authors contributed equally.

[*] Corresponding authors: Alfonso Saiz-Lopez (a.saiz@csic.es); Natalie M. Mahowald (mahowald@cornell.edu)s

Contents**:**

Supplementary Texts S1 to S3.
Supplementary Figures S1 to S14.
Supplementary Tables S1.

Supplementary Texts

S1. Reactive chlorine species - sources and chemistry

Reactive chlorine species discussed in this study refer to those inorganic gas-phase chlorine species (HCl, Cl_2, $ClNO_2$, BrCl, ICl, HOCl, $ClONO_2$, Cl_2O_2, OClO, ClO, and Cl) that actively participates in the tropospheric chemistry, but also to those organic very short-lived chlorine species (CH_2Cl_2, C_2Cl_4, $CHCl_3$, $C_2H_4Cl_2$, C_2HCl_3, CH_2BrCl, $CHBr_2Cl$, $CHBrCl_2$, and CH_2ICl) with a lifetime <180 days that also has some influence on the tropospheric composition. The long-lived halogen species, (e.g., CFC, that are responsible for the stratospheric ozone depletion) (1) are not considered as reactive chlorine species. Reactive chlorine species in the troposphere predominantly originate from three main sectors of sources: natural - sea-salt aerosol is the most critical natural source, anthropogenic – mainly coal burning, solid waste burning, industrial processes, and biomass burning (2).

Figure S1 shows a simplified chemical scheme of the important pathways that are discussed in this work. The first step of tropospheric reactive chlorine is chlorine atom production through photolysis or oxidation (mainly by OH). This chlorine atom will deplete O_3 and consequently reduce the production of OH radical in the troposphere. Conversely, in polluted areas (i.e., VOC and NOx-rich environments) this chlorine atom oxidizes VOCs to produce OH radical. Lastly, this chlorine atom and OH radical are the two main sinks of CH_4 in the troposphere. Therefore, these competing pathways will impact the CH_4 loss and burden. Li et al. (3) found that under RCP6.0 and RCP8.5 cases from the present day to the projected 21st century, unperturbed chlorine levels result in a decrease of O_3 and OH, and consequently increase the abundance of CH_4.

In this study, we vary the molecular chlorine emissions while using RCP6.0 and RCP8.5 as our base cases. As shown in Fig. S2 and S4a, the direct emission of molecular chlorine increases the tropospheric chlorine atom burden. This will lead to a reduction in the abundance of tropospheric OH (Fig. S4b). The contrasting effects of the additional molecular chlorine emission on chlorine atom and OH in the sensitivity scenarios suggest that the additional molecular chlorine emissions increase CH_4 loss through the chlorine channel (Fig. S5c) while decreasing CH_4 loss through the OH channel (Fig. S5b). The combined effects on CH_4 loss (Fig. S5a), lifetime (Fig. S6) and burden (Fig. 1 in the main text) are nonlinear with the increase in Cl burden: in scenarios S10, S40, S60, and S80 the increase in chlorine atom burden leads to decrease in CH_4 loss and increase in CH_4 lifetime and burden; only when the chlorine atom burden is further increased (including the selected scenarios S630, S1250, and S1880), CH_4 loss is increased and CH_4 lifetime and burden is decreased.

S2. RCP6.0 – different future scenario

We have used RCP6.0 scenario to investigate the potential outcome of the same mitigation scenario if future projections will be different (i.e., RCP 6.0 compared to RCP8.5). We used

the same molecular chlorine emission flux as the RCP8.5 S1250 scenario. Fig. S7 shows the methane response to the molecule chlorine addition in RCP6.0 in comparison with RCP8.5. The chlorine atom change in the RCP6.0 mitigation scenario S1250 is slightly higher (37-fold) compared to the mitigation scenario S1250 for RCP8.5 (30-fold). The absolute methane removed is comparable in both cases, but it represents 35% methane removed in the mitigation scenario of RCP8.5 compared to its baseline and 45% methane removed in the mitigation scenario of RCP6.0 compared to its baseline.

Fig. S8 shows that although the mitigation cases have the same addition, the CH_4 consumption pathways are similar to the higher mitigation scenario (S1880), i.e., about 70% of the CH_4 is consumed by chlorine compared to only 30% by OH and other reactions.

Fig. S9 shows the change in CH_4, tropospheric O_3, stratospheric H_2O, and sulphate aerosols for RCP6.0 scenarios, the same as Fig. 3 in the main text. The CH_4 and tropospheric O_3 reduction percentages are higher for the mitigation scenario of RCP6.0 compared to the same mitigation scenario of RCP8.5. The stratospheric H_2O and sulphate aerosols have approximately the same percentage reduction in both mitigation scenarios.

S3. RCP8.5 20 Tg Cl/year scenario – insufficient chlorine addition

Figure 1 in the main text shows the nonlinear response of the system to the addition of molecular chlorine flux. Scenario S10 (plotted in the inset of Fig .1) results in an increase in CH_4 burden and lifetime. We use this case to examine a situation where such a mitigation technique is implemented in the wrong manner.

The outcome of adding 1.2-fold more chlorine atoms to the RCP8.5 case (S10) is not significant, as can be seen in Fig. S10. The CH_4 lifetime is similar to the results from the baseline RCP8.5 case. However, the total lifetime is a little longer with the additional 10 Tg Cl/year molecular chlorine flux. This is a result of the chlorine chemistry consuming the O_3, and in turn the OH while not adding enough chlorine atoms to compensate for this decrease.

The effective radiative forcing in the S10 scenario is similar to that of the baseline RCP8.5. The resulting increase in radiative forcing due to the increase in methane concentration is masked by the decrease in radiative forcing due to the increase in aerosol concentration.

Supplementary Figures

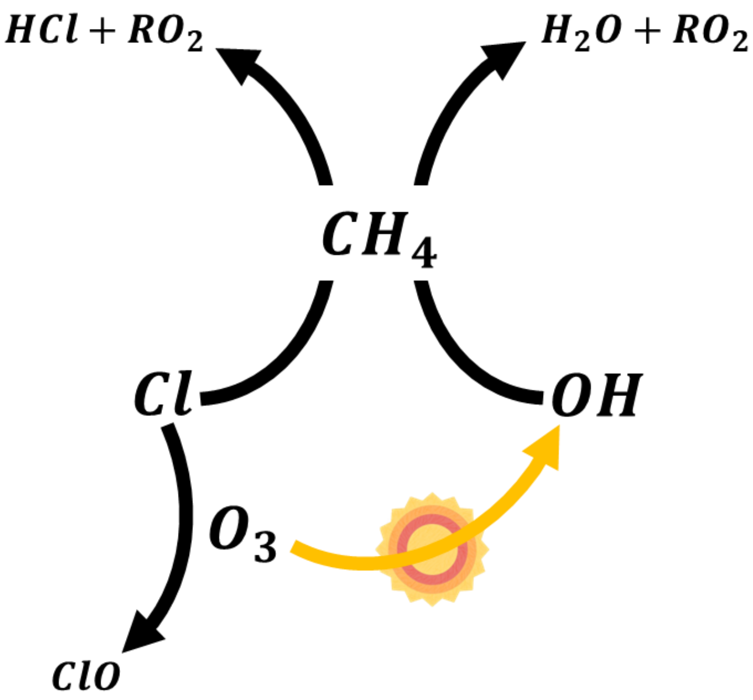

Supplementary Figure S1. Chemical scheme of the main competing reactions discussed in this work.

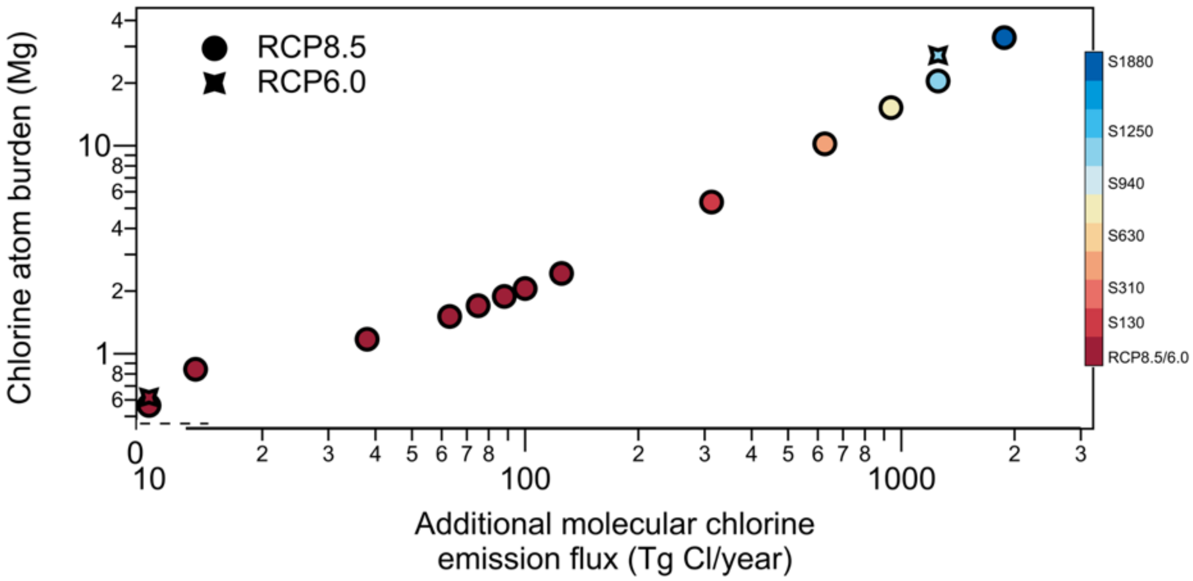

Supplementary Figure S2**.** Tropospheric chlorine atom burden (Mg; averaged between 2020 and 2030) vs additional molecular chlorine emission rate. Please notice that the RCP8.5 and RCP6.0 baseline cases are added at the zero to indicate no addition of molecular chlorine, the rest of the axis is log scaled. The additional molecular chlorine emission flux increases the chlorine atom burden.

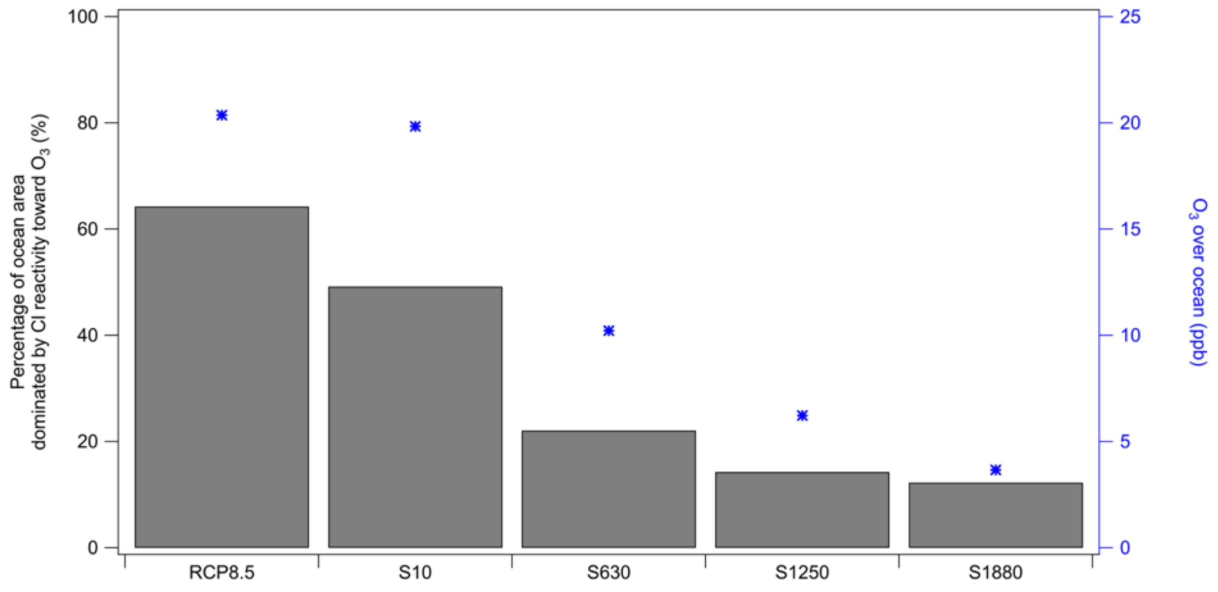

Supplementary Figure S3. Percentage of ocean area dominated by Cl reactivity toward O_3 (grey columns, left axis) and O_3 mixing ratios over the ocean (blue asterisks, right axis) in simulations RCP8.5, S10, S630, S1250, and S1880. In simulation S10, half of the ocean area has higher reactivity of Cl toward O_3 compared to CH_4. Adding more Cl consumes more O_3 over the ocean.

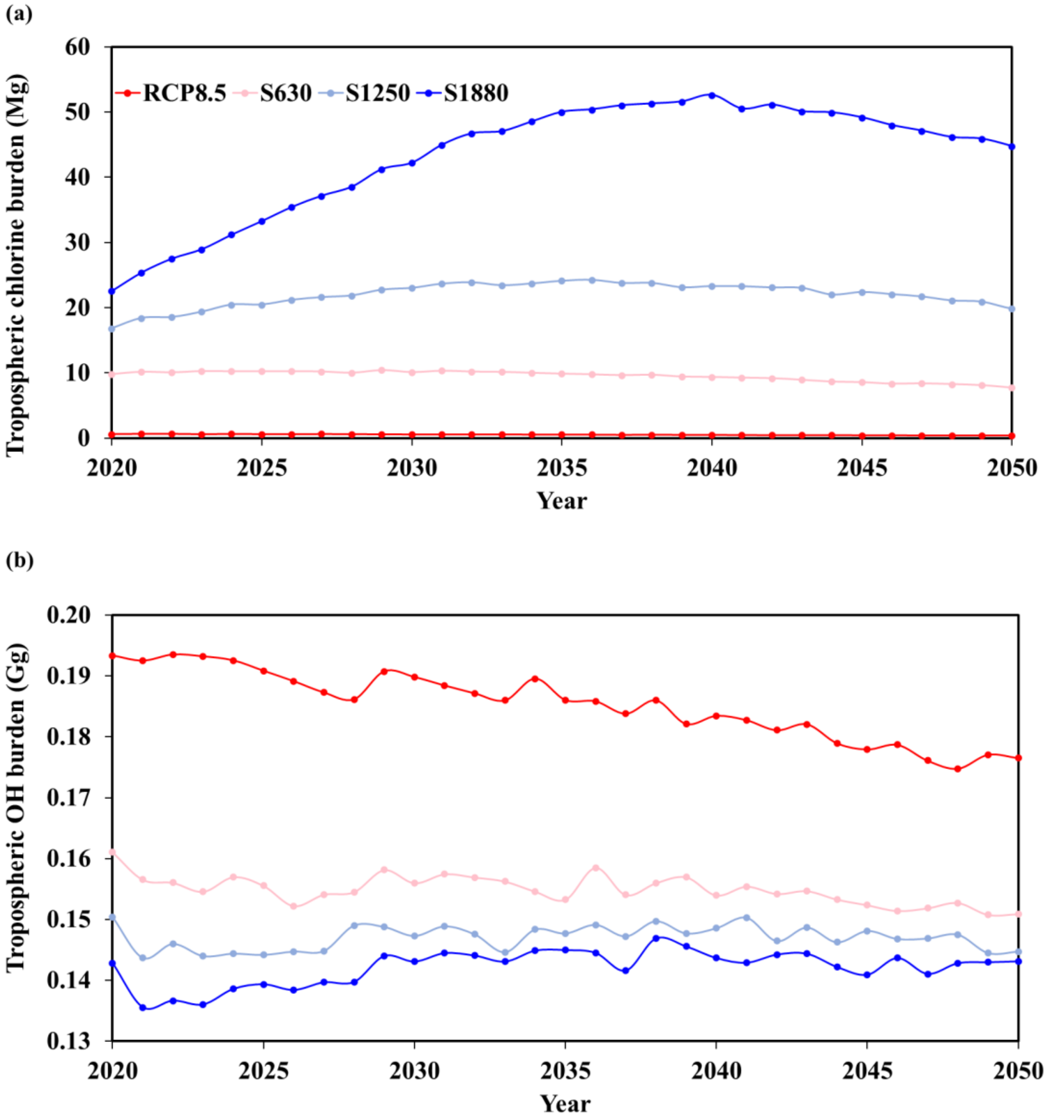

Supplementary Figure S4.Temporal variation of the tropospheric burdens of (a) chlorine atom and (b) OH radical in simulations RCP8.5, S630, S1250, and S1880 (red, RCP8.5; pink: S630; light blue: S1250; dark blue: S1880).

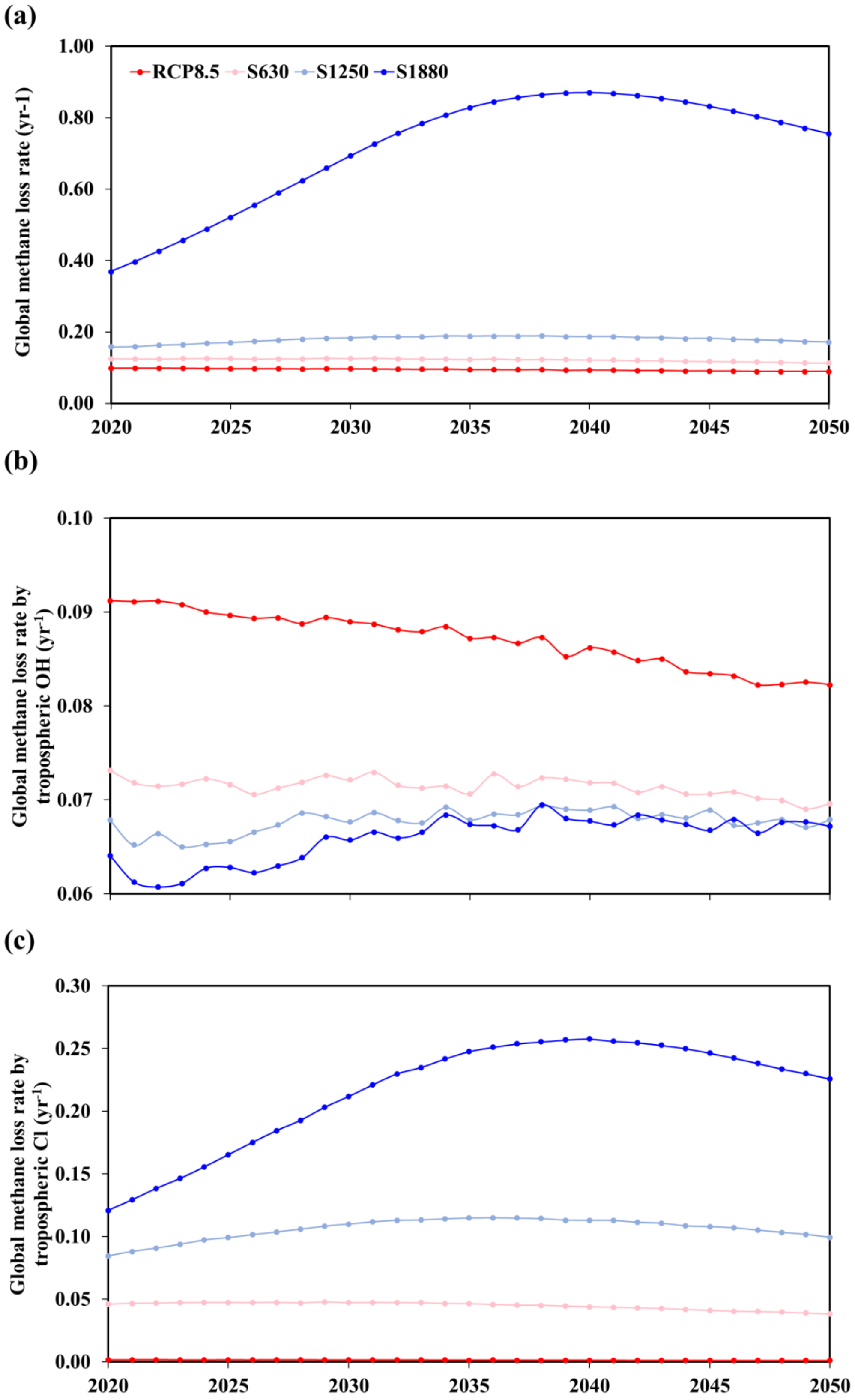

Supplementary Figure S5. Temporal variation of the global CH_4 loss rate by (a) all channels, (b) tropospheric OH, and (c) tropospheric chlorine atom from RCP8.5 case, and S630, S1250, and S1880 scenarios (red, RCP8.5; pink: S630; light blue: S1250; dark blue: S1880).

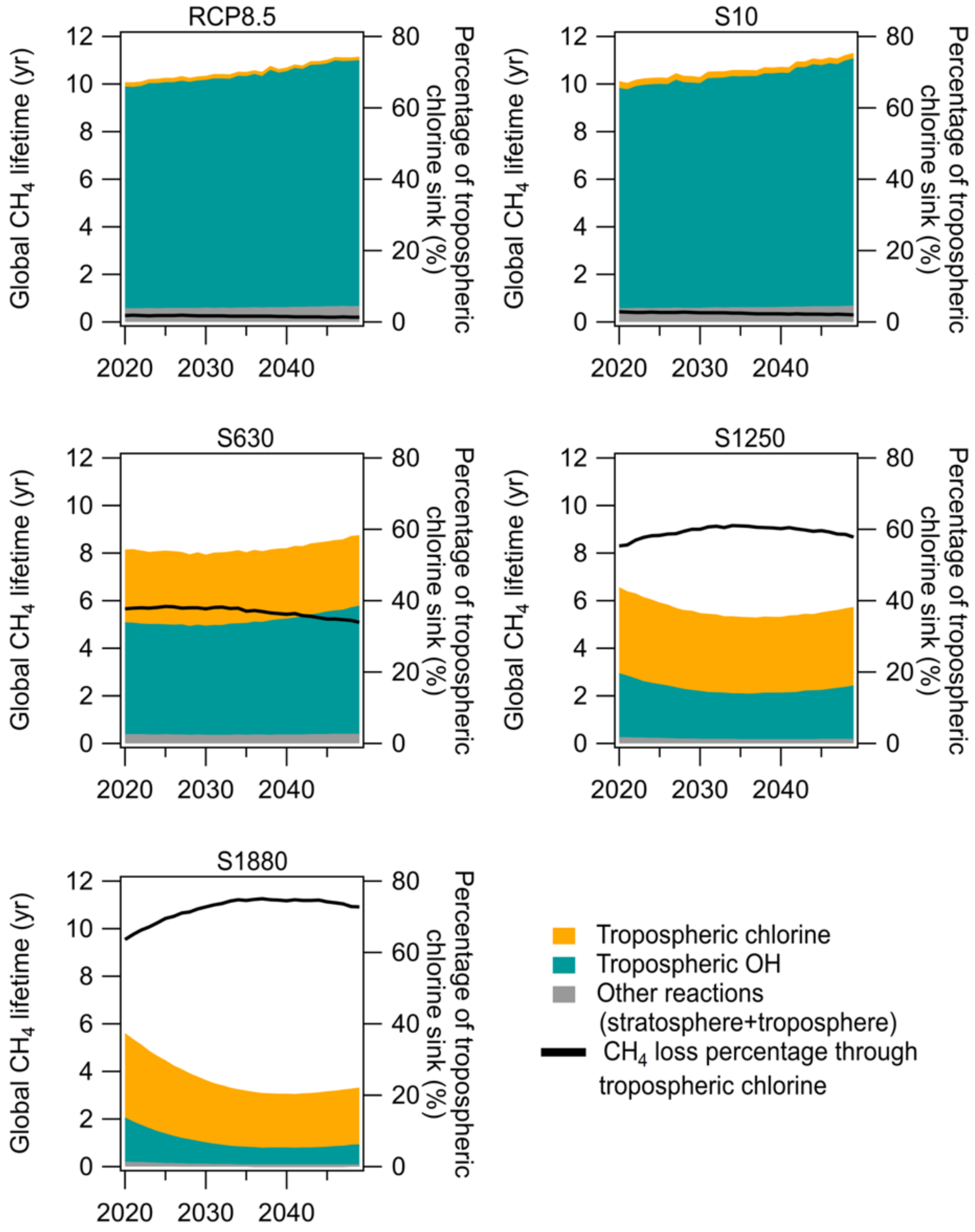

Supplementary Figure S6. Temporal variation of the global CH_4 lifetime for (a) RCP8.5 case, (b) S10, (c) S630, (d) S1250, and (e) S1880 scenarios from 2020 to 2050. The relative contribution of tropospheric OH, tropospheric chlorine, and other chemical loss pathways to the global integrated CH_4 loss is represented by the shading on the left y-axis (tropospheric chlorine-yellow, other reactions-grey and tropospheric OH-teal). The contribution of tropospheric chlorine atom to the total CH_4 life (in percentage) is shown on the right Y-axis. The contribution of each loss channel in the year 2050 for the different scenarios is shown in Fig. 2 in the main text.

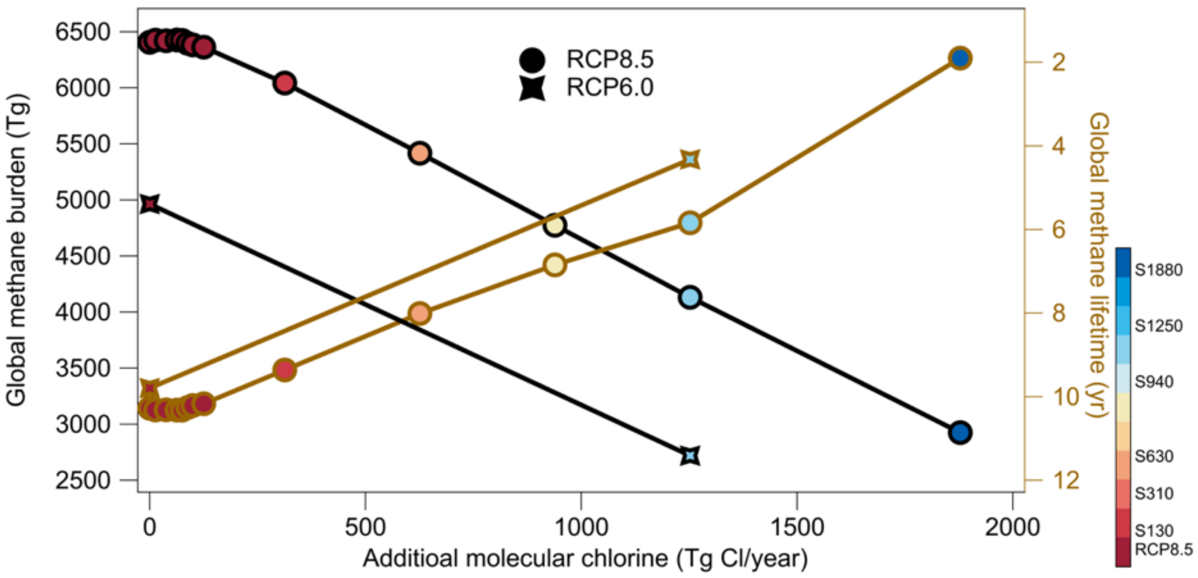

Supplementary Figure S7. CH_4 burden and lifetime vs. additional molecular chlorine flux for the RCP8.5 and RCP6.0 and mitigation scenarios. Same as fig. 1 in the main text. These results are for the year 2030.

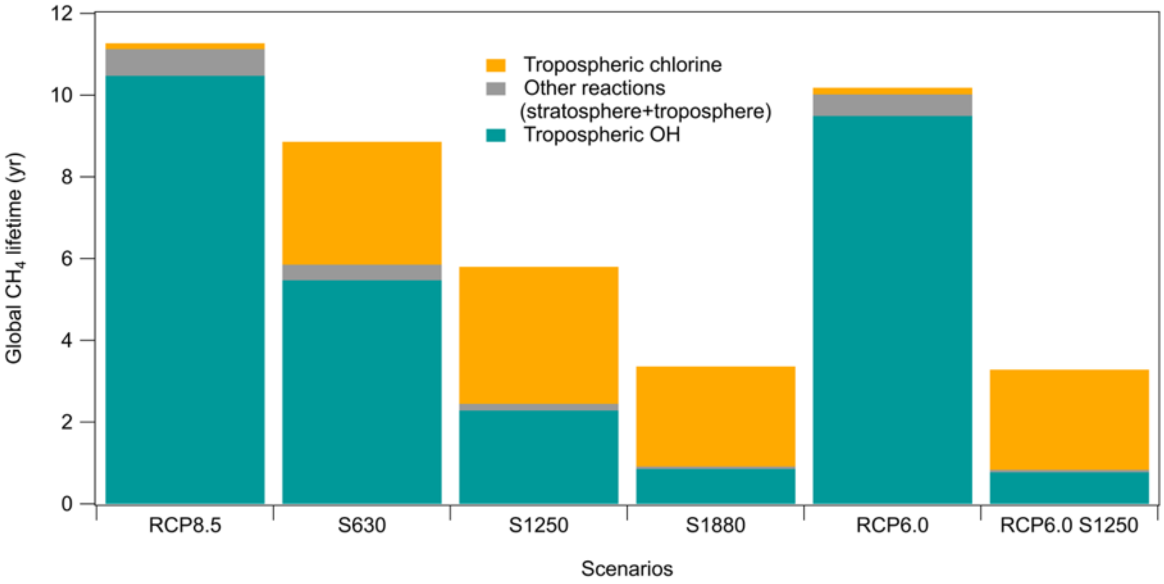

Supplementary Figure S8. Contribution of tropospheric OH, tropospheric chlorine, and other chemical loss pathways to the global integrated CH_4 lifetime in the year 2050 for RCP8.5, RCP6.0 and mitigation scenarios. The relative contribution of tropospheric OH, tropospheric chlorine, and other chemical loss pathways to the global integrated CH_4 loss is represented by the shading (tropospheric chlorine-yellow, other reactions-grey and tropospheric OH-teal). Same as Fig. 2 in the main text. The same mitigation scenario results in higher methane loss driven by chlorine for the RCP6.0 S1250 compared to the RCP8.5 S1250.

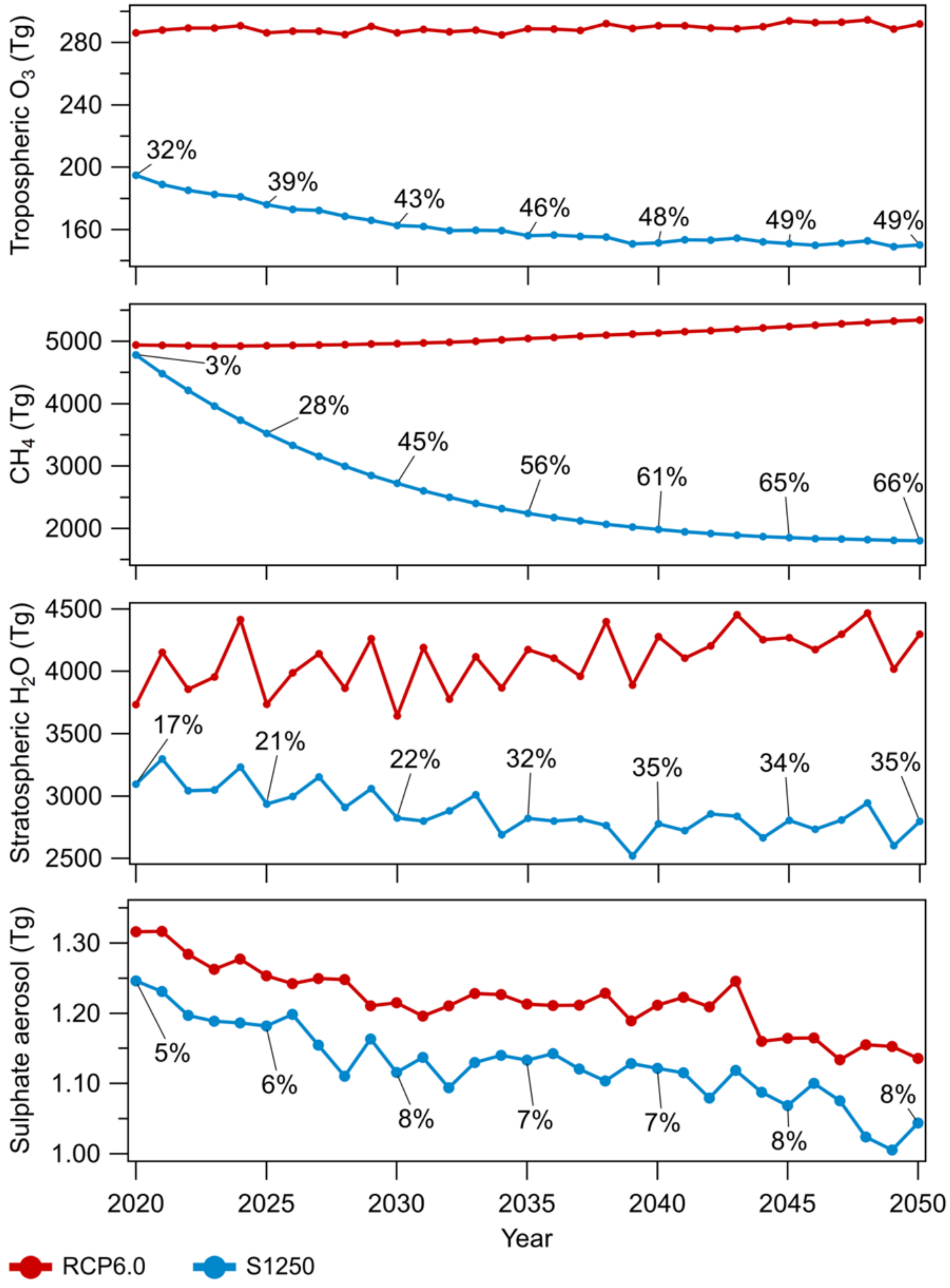

Supplementary Figure S9. Short-lived climate forcers global burdens. Top to bottom panels – CH_4, Tropospheric O_3, Stratospheric H_2O, and sulphate aerosol (red, RCP6.0; light blue, RCP6.0 S1250). The percentage reduction compared to RCP6.0 is shown as numbers on each plot. Tropospheric O_3 and CH_4 are reduced by similar percentage as the RCP8.5 S1880 scenario. Same as the RCP8.5, the percentage change stabilizes after 2035 for all SLCFs.

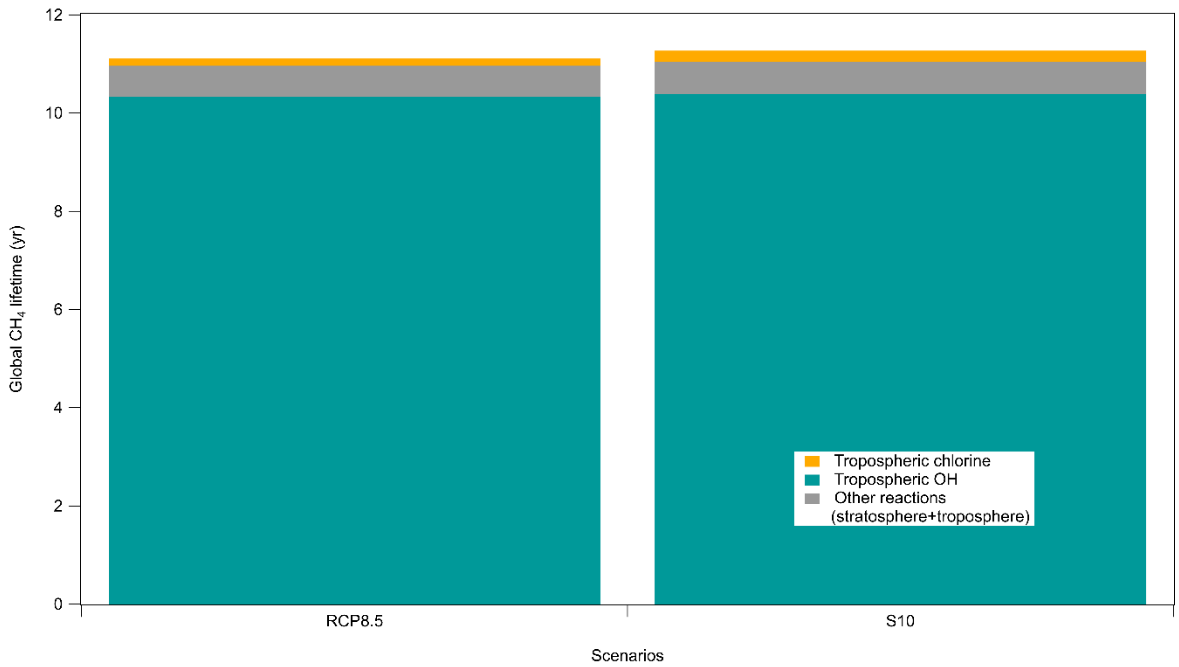

Supplementary Figure S10. Contribution of tropospheric OH, tropospheric chlorine, and other chemical loss pathways to the global integrated CH_4 lifetime in the year 2050 for RCP8.5, and scenario S10. The relative contribution of tropospheric OH, tropospheric chlorine, and other chemical loss pathways to the global integrated CH_4 loss is represented by the shading (tropospheric chlorine-yellow, other reactions-grey and tropospheric OH-teal).

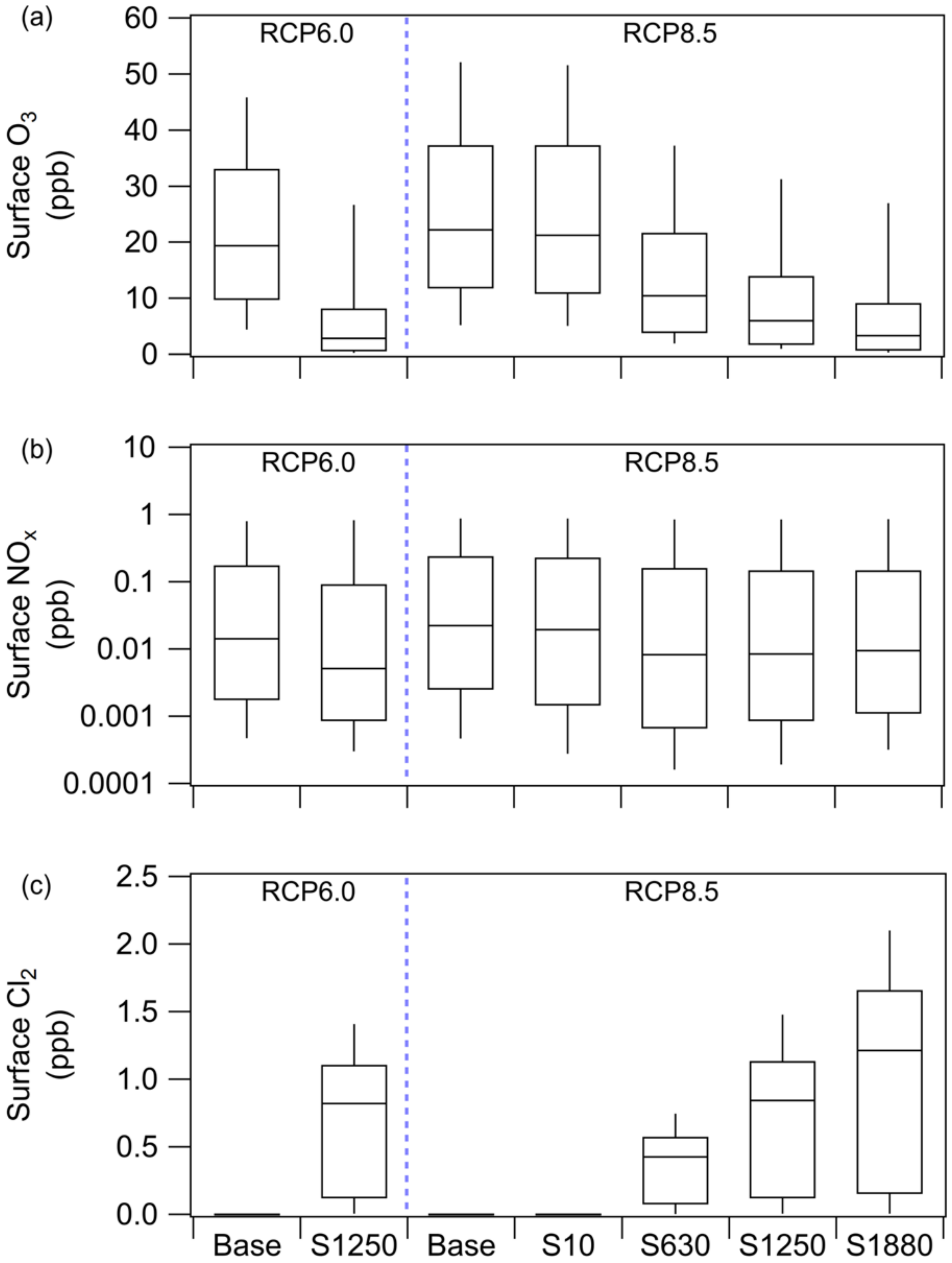

Supplementary Figure S11. Global surface mixing ratios of (a) O_3, (b) NO_x (log scaled), and (c) Cl_2 in the year 2050 for RCP8.5, RCP6.0 and mitigation scenarios. In the mitigation scenario (S1250) based on RCP6.0, the surface mixing ratios are similar to those in RCP8.5 S1880.

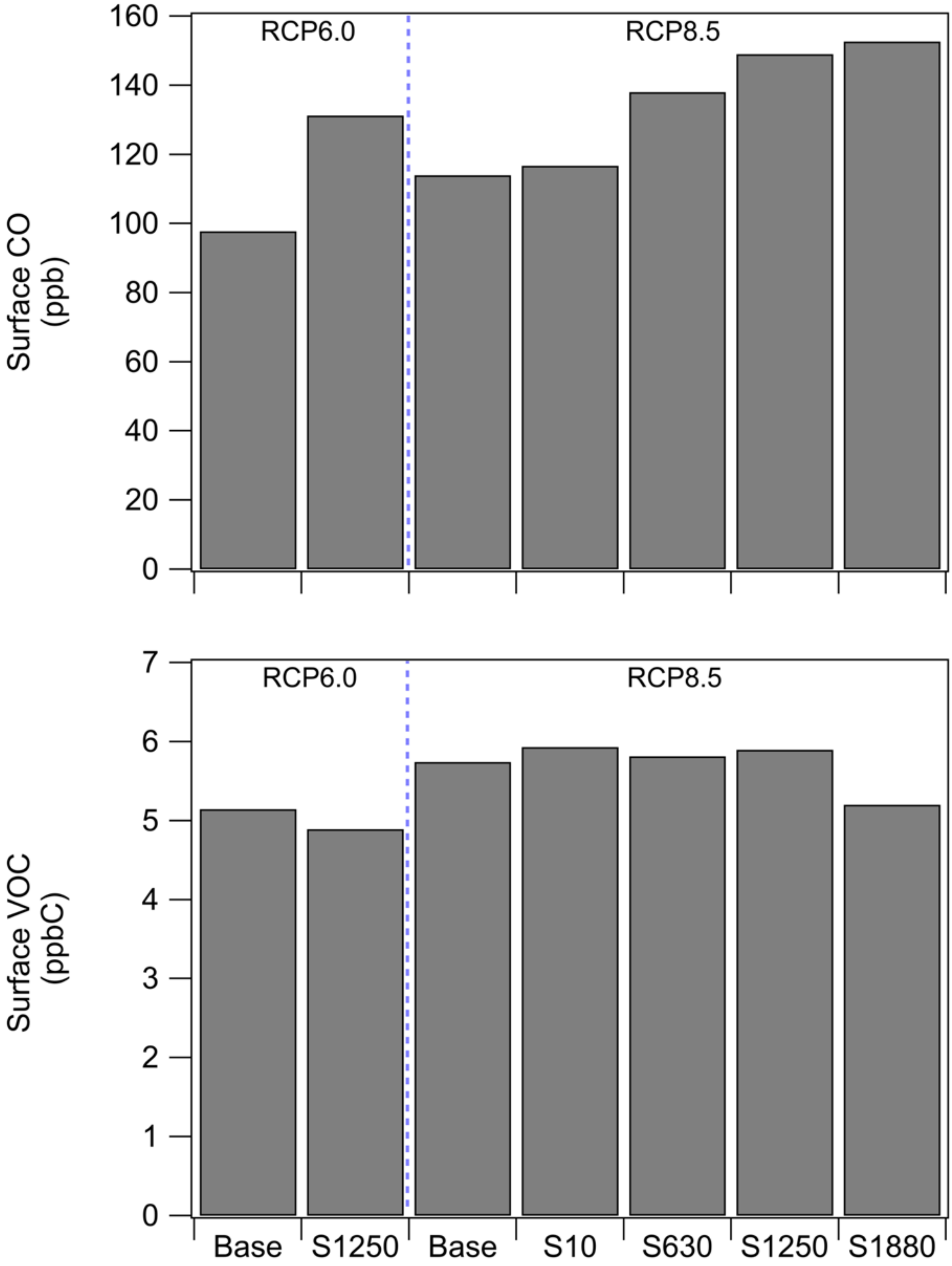

Supplementary Figure S12. Global surface mixing ratios of CO (top) and VOC (bottom) in the year 2050 for RCP8.5, RCP6.0 and mitigation scenarios.

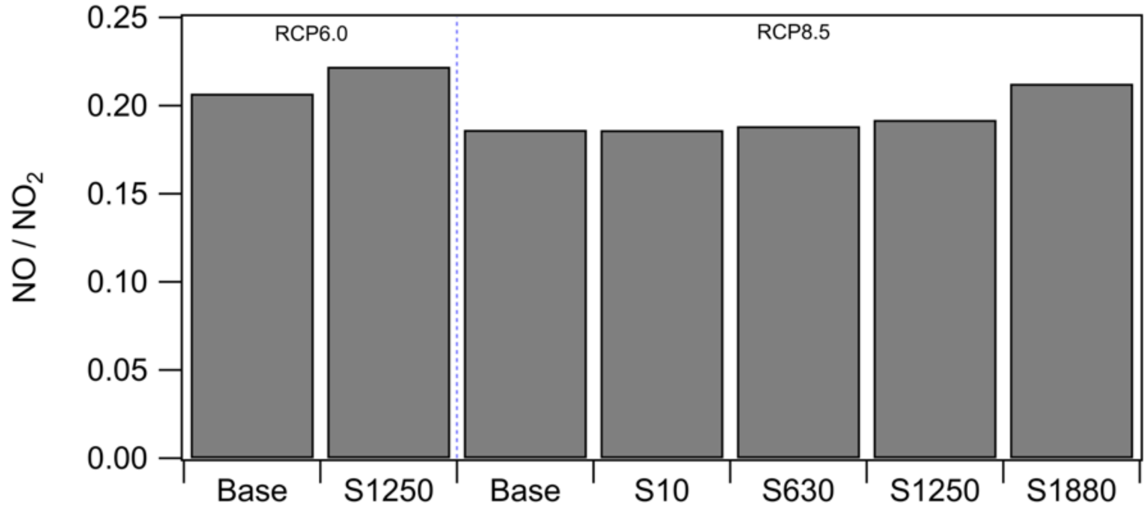

Supplementary Figure S13. Global NO to NO_2 ratio in the year 2050 for RCP8.5, RCP6.0 and mitigation scenarios.

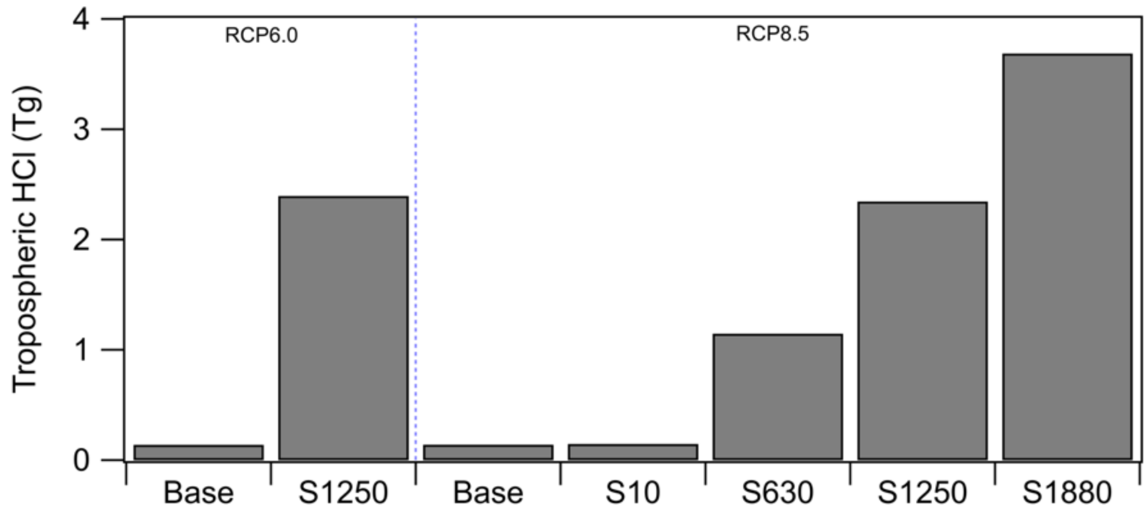

Supplementary Figure S14. Tropospheric HCl burden in the year 2050 for RCP8.5, RCP6.0 and mitigation scenarios.

Supplementary Tables

Supplementary Table S1. CESM simulation designs.

Simulations	Addition molecular chlorine flux over oceanic surface		Increase in chlorine atom burden	Simulation period
	Tg Cl/year	molecule/cm^2/s		
RCP8.5	-	-	-	30 years (2020-2050)
S10	13	1E9	1.2-fold	30 years (2020-2050)
S40	38	3E9	1.7-fold	10 years (2020-2030)
S60	63	5E9	2.2-fold	10 years (2020-2030)
S80	75	6E9	2.5-fold	10 years (2020-2030)
S90	88	7E9	2.8-fold	10 years (2020-2030)
S100	100	8E9	3.1-fold	10 years (2020-2030)
S130	125	1E10	3.6-fold	10 years (2020-2030)
S310	313	2.5E10	7.9-fold	10 years (2020-2030)
S630	626	5E10	15-fold	30 years (2020-2050)
S940	939	7.5E10	23-fold	30 years (2020-2050)
S1250	1252	1E11	30-fold	30 years (2020-2050)
S1880	1878	1.5E11	50-fold	30 years (2020-2050)
RCP6.0	-	-	-	30 years (2020-2050)
RCP6.0 S1250	1252	1E11	37-fold	30 years (2020-2050)
